# Effect of hydroalcoholic extract of *Sphaeranthus indicus* against experimentally induced anxiety, depression and convulsions in rodents

**DOI:** 10.4103/0974-7788.64412

**Published:** 2010

**Authors:** Varsha J. Galani, Bharatkumar G. Patel

**Affiliations:** *Department of Pharmacology, A. R. College of Pharmacy and G. H. Patel Institute of Pharmacy, Vallabh Vidyanagar - 388120, Gujarat, India*; 1 *Department of Pharmacology, Sardar Patel University, Vallabh Vidyanagar - 388120, Gujarat, India*

**Keywords:** Elevated plus maze test, forced swim test, maximal electroshock-induced convulsions, open field test, pentylenetetrazole-induced convulsions, *Sphaeranthus indicus*

## Abstract

To investigate the effects of a hydroalcoholic extract of the *Sphaeranthus indicus* (SIE) against experimentally induced anxiety, depression and convulsions in rodents. The SIE (100, 200, 500 mg/kg, *p.o*.) was used in elevated plus maze, open field, forced swimming, and tail suspension tests in mice. The same doses were also used to evaluate its anticonvulsant effect on pentylenetetrazole (PTZ)-induced convulsions in mice and maximal electroshock (MES)-induced convulsions in rats. SIE was found to increase the number of entries and the time spent in the open arms of the maze at a dose of 100 mg/kg, *p.o*., indicating its anxiolytic activity. On the other hand, higher doses of SIE (200 and 500 mg/kg, *p.o*.) decreased open arm entries and time spent in the open arms of the maze in the elevated plus maze test indicating an absence of anxiolytic activity. However, this effect could have been related to a decrease in the locomotor activity of the mice and not to an anxiogenic effect, as indicated by the reduction in the total number of entries in the elevated plus maze. SIE also (at doses of 200 and 500 mg/kg, *p.o*.) decreased locomotor activity but did not affect emotional activity parameters in the open field test, suggesting a possible central nervous depressant activity. SIE also increased the immobility time in the forced swimming test at an oral dose of 500 mg/kg but did not significantly modify the activity in the tail suspension test. SIE protected rats against MES-induced convulsions and mice against PTZ-induced convulsions. *Sphaeranthus indicus* demonstrated anxiolytic, central nervous depressant, and anticonvulsant activities in rodents, thus supporting the folk medicinal use of this plant in nervous disorders.

## INTRODUCTION

*Sphaeranthus indicus* Linn. (*Asteraceae*), commonly known as '*Gorakhmundi*,' is a highly branched herb that is found to be distributed throughout the plains in India in wet places. In folk medicine, the entire herb is used for insanity, epileptic convulsions, vomiting, and hemicranias,[1] and is valued as an aphrodisiac and nervine tonic.[2,3] Previous phytochemical studies have reported the presence of a sesquiterpene lactone,[4] a steroid,[5] a flavanoid,[6] an essential oil,[7] and eudesmanolides[4,8] in *S. indicus*. The herb has been reported to have antibacterial, antifungal,[9] immunomodulatory,[8] antioxidant,[10] and hypoglycemic[11] activities, while neuroleptic[12] and anxiolytic activity[13] has been reported for flowers of this plant. These reported activities confirm that the herb of *S. indicus* is able to modulate the physiology of the central nervous system. Various activities of the entire herb have been reported but no scientific data are available for the central nervous actions of the entire herb of *S. indicus*. Hence, the present study was designed to evaluate the effects of a hydroalcoholic extract of *S. indicus* on experimentally induced anxiety, depression and convulsions in rodents.

## MATERIALS AND METHODS

### Animals

Albino Wistar mice (25-30 g) and rats (200-250 g) of either sex were bred in the Central Animal House facility of the institute and the animals were housed under standard conditions. Briefly, they were maintained on a 12 h light/dark cycle and given free access to food and water up to the time of experimentation. The animals were acclimatized to the laboratory environment for one hour before the experiments. Animals were randomly distributed into groups of ten animals each. All experiments were conducted during the light period (08.00-16.00 h). All the protocols were approved by the Institutional Animal Ethics Committee (IAEC) and conducted according to the guidelines of CPCSEA (Committee for the Purpose of Control and Supervision of Experiments on Animals).

### Plant material and preparation of extract

Fresh, fully grown flowering herbs of *Sphaeranthus indicus* were collected from the campus of our institute and authenticated by a taxonomist in the Department of Bioscience, Sardar Patel University, Vallabh Vidyanagar, Gujarat. A specimen of the plant has been kept in the herbarium of our institute (Voucher No. ARGH7). The plant material was completely dried under shade and powdered. The powdered material was extracted exhaustively with 50% ethanol by maceration for two days at room temperature with occasional shaking. The crude (hydroalcoholic) extract was filtered and dried under reduced pressure at 40°C (yield: 11.1% w/w).

### Preliminary phytochemical screening

The hydroalcoholic extract of the *Sphaeranthus indicus* herb was tested for the presence of carbohydrates, proteins, alkaloids, flavonoids, glycosides, saponins, tannins, and essential oils using standard procedures.[14]

### Experimental protocol

A freshly prepared aqueous solution of the dried extract of the *S. indicus* herb (SIE) was suitably diluted before administration to test animals. The vehicle, distilled water (10 mL/kg, *p.o*.), was administered to control animals. Diazepam was used as a reference drug for the elevated plus maze test (1 mg/kg, *i.p*.), the open field test (1 mg/kg, *i.p*.), and to study pentylenetetrazole-induced convulsions (4 mg/kg, *i.p*.). Imipramine (10 mg/kg, *i.p*.) was used as a reference drug to study antidepressant action in the forced swimming and tail suspension tests. Phenytoin (25 mg/kg, *i.p*.) was used as a reference drug for maximal electroshock-induced convulsions. For the present experimental study, animals were divided into five groups with each group consisting of ten animals. Group 1 served as a control group and received distilled water (vehicle; 10 mL/kg, *p.o*.), groups 2-4 served as test groups receiving SIE (100, 200, and 500 mg/kg, *p.o*) whereas group 5 served as a positive control and received the reference drug. The animals were subjected to various behavioral tests an hour after oral and 30 min after intraperitoneal administration.

### Elevated plus maze test

The elevated plus maze used in this study was modified from the one described by Lister (1987).[15] The plus maze consisted of two opposite arms, 25 cm × 5 cm, crossed with two closed arms of the same dimensions with 30 cm-high walls. The arms were connected with a central square, 7.5 cm × 7.5 cm, to give the apparatus the shape of a plus sign. The whole apparatus was elevated 25 cm above the floor in a dimly illuminated room. When exposed to this novel maze alley, the animals experienced an approach-avoidance conflict, which was stronger in the open arms than in the enclosed arms. Animals were placed individually in the center of the maze, facing a closed arm, after which the number of entries and time spent in the enclosed and open arms were recorded during the next five minutes. An arm entry was defined as the presence of all four feet in that particular arm. A selective increase in open arm exploration was observed as a consequence of anxiolytic drug administration. The maze was cleaned after each trial to remove any residue or odor of the animals.

### Open field test

The apparatus consisted of a dimly lit area of 96 × 96 cm that was divided into 16 squares. Mice were placed individually in one corner of the apparatus and observed for a period of three minutes to detect the number of peripheral squares crossed, number of central squares crossed, periods of immobility, as well as the numbers of rearings and fecal pallets.[16]

### Forced swimming test

Mice were made to swim individually in a polypropylene vessel (30 × 15 × 30 cm) with a water level of 15 cm at 25 ± 2°C. The mouse was initially allowed to swim for 10 min after which the total periods of immobility characterized by a complete cessation of swimming with the head just floating above water level, was determined during the subsequent five-minute period.[17]

### Tail suspension test

This test is a variant of the forced swimming test in which immobility was induced by suspending a mouse by its tail. Mice were hung on a wire individually in an upside-down posture so that their nostrils touched the water surface in a container. After initial vigorous movements, the mice assumed immobility during a five-minutes observation period and these periods of immobility were recorded.[18]

### Maximal electroshock-induced convulsions

Albino rats of either sex were given supramaximal electroshock of 150 mA for a period of 0.2 seconds through a pair of corneal electrodes by using an electroconvulsiometer (Techno., India). Animals showing positive hind limb extensor responses during prescreening were selected. These animals were treated as per the experimental protocols described above. The test was repeated after drug treatments on the next day. The rats were manually restrained and released immediately after the electrically induced convulsions and the seizure was observed throughout its course after stimulation. The severity of convulsions was assessed by the duration of tonic flexion, tonic extensor, clonus, and stupor phase for each animal. The duration of each phase for each animal (sec) was measured by using a stopwatch. The criterion for anticonvulsant activity and protection against MES-induced seizures was the abolition of hind limb tonic extension (HLTE), which was taken as the end point of the test.[19]

### Pentylenetetrazole-induced convulsions

The test was conducted in mice one hour after vehicle (1 mL/kg, *p.o*.) or SIE (100, 200, and 500 mg/kg, *p.o*.) or diazepam (4 mg/kg, *i.p*.) treatment. Pentylenetetrazole (PTZ) was injected intraperitoneally (50 mg/kg) into groups of mice[20] which were observed for the incidence of convulsions, latency to first convulsion and duration of convulsions.

### Statistical analysis

The data were expressed as mean ± S.E.M. Statistical analysis was done using one-way analysis of variance (ANOVA) followed by Dunnett's test. Results were considered significant at *P* < 0.05.

## RESULTS

### Preliminary phytochemical screening

Phytochemical screening revealed the presence of carbohydrates, proteins, flavonoids, triterpenes, steroids, saponins, tannins, coumarins and essential oil in the hydroalcoholic extract of the *S. indicus* herb.

### Effect on elevated plus maze test

Results of the effects of SIE on entries and time spent in both the arms (open and enclosed) of the elevated plus maze are shown in Table 1. In this test, the number of entries and time spent in the open arms were considered for the analysis of anxiolytic activity and the total number of entries in both the arms (enclosed and open arms) was considered for the evaluation of locomotor activity of animals. SIE (100 mg/ kg, *p.o*.) was found to significantly ( *P* < 0.05) increase the number of entries of the mice and the time spent in the open arms compared to the control animals. This result demonstrates the anxiolytic activity of SIE at the 100 mg/kg oral dose. At higher oral doses (200 and 500 mg/kg), SIE did not change the number of entries or time spent in the open arms. However, the total time spent in the closed arm was found to be significantly increased ( *P* < 0.05) with 200 and 500 mg/kg oral doses of SIE. The total numbers of entries were however, found to be significantly reduced at 200 and 500 mg/kg oral doses of SIE, indicating a reduction in locomotor activity of the mice. The total time spent in the arms was not changed by any dose of SIE. The positive control, Diazepam (1 mg/kg, *i.p*.), was found to significantly ( *P* < 0.05) increase the number of entries in the open arms as well as the duration of stay in the open arms, indicating anxiolytic activity. Diazepam also increased the total number of entries in the elevated plus maze.

**Table 1:** Effect of hydroalcoholic extract of *S. indicus* on elevated plus maze test in mice

Treatment	Dose(mg/kg)	Time spent on (sec)		Enteries on	
		Enclosed arm	Open arms	Enclosed arm	Open arm
Control	-	197.7 ± 14.4	28.3 ± 6.50	6.8 ± 0.58	1.5 ± 0.32
SIE	100	189.8 ± 15.7	54.5 ± 4.29*	3.9 ± 0.48*	3.7 ± 0.51*
SIE	200	279.9 ± 14.5*	35.8 ± 2.97	3.0 ± 0.47*	1.9 ± 0.43
SIE	500	280.3 ± 23.5*	33.9 ± 3.17	3.1 ± 0.39*	2.0 ± 0.44
Diazepam	1	145.7 ± 11.4	41.4 ± 3.73	9.5 ± 0.57*	4.9 ± 0.57*

Values are expressed as mean ± SEM (*n* = 10). One-way ANOVA followed by Dunnett's test

* *P* < 0.05 when compared with control group

### Effect on open field test

The overall results of the open field test are summarized in Table 2. As expected, when released into the open field, the control animals started moving along the walls, with their initial exploration being limited mostly to the peripheral squares; the inner squares were rarely explored. Therefore, peripheral square entry in control animals was high and central square entry was low. Mice treated with a 100 mg/kg oral dose of SIE did not show significantly altered peripheral square crossing, central square crossing, or immobility time in this test. At oral doses of 200 and 500 mg/kg, SIE was seen to produce significant reduction in the number of peripheral squares and central squares crossed by mice in the open field test. Statistical analysis revealed that the oral dose of 500 mg/ kg of SIE significantly increased the immobility time of mice. Mice treated with the SIE (100, 200, and 500 mg/ kg, *p.o*.) and diazepam (1 mg/kg, *i.p*.) did not show any statistically significant alteration in their rearing and fecal dropping behaviors. Diazepam-treated animals showed increased numbers of peripheral square crossing and central square crossing, but these differences were not found to be statistically significant. Also, diazepam treatment did not significantly increase the immobility time of mice in the open field test.

**Table 2:** Effect of hydroalcoholic extract of *S. indicus* on open field test in mice

Treatment	Dose (mg/kg)	Peripheral square crossed (number)	Central square crossed (number)	Rearing (counts)	Fecal dropping (counts)	Time of immobility (sec)
Control	-	28.8 ± 5.5	3.2 ± 1.4	26.3 ± 9.3	0.6 ± 0.4	15.0 ± 7.51
SIE	100	20.4 ± 3.71	2.5 ± 0.75	44.2 ± 10.7	0.3 ± 0.29	51.2 ± 8.81
SIE	200	9.2 ± 3.41*	0.5 ± 0.47*	48.7 ± 8.2	1.5 ± 0.41	63.4 ± 20.3*
SIE	500	5.7 ± 2.34*	0.8 ± 0.48*	41.4 ± 9.8	1.3 ± 0.32	47.4 ± 7.18
Diazepam	1	36.2 ± 5.28	4.1 ± 0.49	5.6 ± 1.68	0.6 ± 0.21	59 ± 9.17

Values are expressed as mean ± SEM (*n* = 10). One-way ANOVA followed by Dunnett's test

* *P* < 0.05 when compared with control group

### Effect on forced swimming and tail suspension tests

The effect of SIE on the forced swimming test was measured by the time of immobility of the mice during the observation period. In the forced swimming test, mice treated with the oral dose of 500 mg/kg of SIE showed a significant increase in the immobility time ( *P* < 0.05) when compared to control mice. In contrast, 100 and 200 mg/kg oral doses of SIE did not result in any significant increase in the immobility time of mice. None of the tested doses of SIE (100, 200 and 500 mg/ kg, *p.o*.) were found to significantly prolong the immobility time of the mice in the tail suspension test [Table 3].

**Table 3:** Effect of hydroalcoholic extract of *S. indicus* in forced swimming and tail suspension tests in mice

Treatment	Dose (mg/kg)	Immobility time in forced swimming test (sec)	Immobility time in tail suspension test (sec)
Control	-	137.4 ± 937	68.5 ± 10.86
SIE	100	149.2 ± 9.14	68.6 ± 11.78
SIE	200	151.4 ± 10.7	69.4 ± 10.69
SIE	500	191.9 ± 23.8*	81.6 ± 9.69
Imipramine	10	71.5 ± 11.45*	26.8 ± 6.03*

Values are expressed as mean ± SEM (*n* = 10). One-way ANOVA followed by Dunnett's test

* *P* < 0.05 when compared with control group

### Effect on maximal electroshock-induced convulsions

The effects of SIE on the flexion and extension phases of maximal electroshock-induced convulsions in rats are shown in Table 4. At the highest tested oral dose (500 mg/kg), SIE was found to significantly decrease the duration of the hind limb tonic extensor phase in MES-induced seizures whereas the lower doses (100 and 200 mg/kg, *p.o*.) did not give any protection. Rats treated with phenytoin (25 mg/kg) were found to be completely protected from MES-induced convulsions, as demonstrated by the absence of all the phases of convulsion.

**Table 4:** Effect of hydroalcoholic extract of *S. indicus* on maximum electroshock-induced convulsions in rats

Treatment	Dose (mg/kg)	Incidence	Phases of MES-induced seizures (sec)			
			Tonic fl exion	Hind limb tonic extension	Clonic	Stupor
Control	-	10/10	4.2 ± 0.42	9.2 ± 0.97	13.9 ± 2.57	36.1 ± 5.32
SIE	100	10/10	3.2 ± 0.31	9.1 ± 1.01	13.5 ± 2.07	25.3 ± 356
SIE	200	10/10	3.4 ± 0.25	7.6 ± 1.42	10.1 ± 1.09	31.2 ± 6.04
SIE	500	10/10	3.9 ± 0.26	5.3 ± 0.81*	11.5 ± 1.49	25.7 ± 4.68
Phenytoin	25	0/10	-	-	-	-

Values are expressed as mean ± SEM (*n* = 10). One-way ANOVA followed by Dunnett's test

* *P* < 0.05 when compared with control group

### Effect on pentylenetetrazole-induced convulsions

All the oral doses (100, 200, and 500 mg/kg) of SIE were found to produce significant ( *P* , 0.05) and dose-dependent reduction in the duration of the first clonic convulsion in mice [Table 5]. However, SIE treatment (100, 200, and 500 mg/ kg, *p.o*.) did not significantly affect the latency of onset of pentylenetetrazole-induced convulsions in mice; no change was observed in the incidence of convulsion and mortality. As expected, diazepam (4 mg/kg, *i.p*.)-treated mice did not have any convulsive episode or show any mortality when treated with pentylenetetrazole, the results demonstrated 100% protection of the test animals as compared to the control.

**Table 5:** Effect of hydroalcoholic extract of *S. indicus* on pentylenetetrazole (50 mg/ kg, *i.p*.)-induced seizures in mice

Treatment	Dose (mg/kg)	Pentylenetetrazole-induced generalized clonic seizures		
		Incidence	Latency (sec)	Duration (sec)
Control	-	10/10	116.7 ± 13.6	10.4 ± 0.54
SIE	100	10/10	129.5 ± 15.2	7.2± 0.44*
SIE	200	10/10	139.8 ± 12.8	6.8 ± 0.31*
SIE	500	8/10	183.3 ± 18.9	6.6 ± 0.57*
Diazepam	4	0/10	-	-

Values are expressed as mean ± SEM (*n* = 10). One-way ANOVA followed by Dunnett's test

* *P* < 0.05 when compared with control group

## DISCUSSION

The present study evaluated the effects of a hydroalcoholic extract of the *S. indicus* herb on experimentally induced anxiety, depression, and convulsions. The elevated plus maze is considered to be an etiologically valid animal model of anxiety. The number of entries and time spent in the open arms have been found to be increased by anxiolytics and reduced by anxiogenic agents.[21] Anxiolytic activity was observed for an oral dose of 100 mg/kg dose of SIE. However, at higher oral doses of 200 and 500 mg/kg doses of SIE, significant reduction in the number of entries and time spent in the open arms were observed. Furthermore, no anxiolytic or anxiogenic effects were observed with SIE treatment under these conditions as locomotor activity was impaired after SIE administration. Thus, there is a dose-dependency for the effects observed for SIE in this study. As expected, diazepam reduced the animal's natural aversion to the open arms and promoted maze exploration. Literature reports describe the action of benzodiazepines, such as diazepam, as anxiolytics (at low doses) and as anticonvulsants, also producing sedation and myorelaxant effects at higher doses.[22,23] Reduction in the locomotor activity by the hydroalcoholic extract of *S. indicus* in the elevated plus maze test may be correlated with central nervous depression.[24] The open field test is a paradigm used for evaluating the effect of drugs on gross general behavior and is used to measure the level of nervous excitability. [25] When removed from their acclimatized home cages and placed in a novel environment, animals express their anxiety and fear by showing decreased ambulation and exploration, immobilization or freezing, and reduction in normal rearing and grooming behavior. Increased micturation and defecation due to augmented autonomic activity is also observed. These paradigms are attenuated by classical anxiolytics and potentiated by anxiogenic agents.[16] The hydroalcoholic extract of *S. indicus* was seen to increase immobility time and decrease peripheral square movements; the observed decrease in central square movements could be due to impairment with locomotor activity. The decrease in locomotor activity in the open field test of mice treated with hydoralcoholic extract of *S. indicus*, produces more evidence for its central nervous depressant activity.[24] Emotional activity parameters such as rearing and fecal dropping in mice were not significantly affected by treatment with the hydroalcoholic extract of *S. indicus*.

Forced swimming and tail suspension tests are widely used to screen new antidepressant drugs.[17,26] The results of the forced swimming and tail suspension tests showed a significant increase in the immobility time upon treatment with the hydroalcoholic extract of *S. indicus*. Thus, the overall results seem to be predictive for the central nervous depressant action of this hydroalcoholic extract of *S. indicus*.

The criterion for anticonvulsant activity and protection against maximal electroshock-induced convulsions was the abolition of hind limb tonic extension. Significant reduction of hind limb tonic extensor phase in rats by prior administration of the hydroalcoholic extract of *S. indicus* (500 mg/kg, *p.o*.) may relate with its anticonvulsant action. Pentylenetetrazole is the most frequently used substance and an acute experimental model in the preliminary screening of potential anticonvulsant drugs. The induction of convulsions by pentylenetetrazole is attributed to the repression of the function of gamma-aminobutyric acid type A (GABA_A_ ) receptor Cl^–^channel.[27] Benzodiazepine agonists, like diazepam, are positive allosteric modulators of GABA-mediated neurotransmission in the central nervous system. Anticonvulsant potential of the hydroalcoholic extract of *S. indicus* was also demonstrated by the partial protection from pentylenetetrazole-induced convulsions.

The efficacy of most herbal remedies is attributed to the combination of various active principles. The observed pharmacological actions of the hydroalcoholic extract of *S. indicus* may be due to the presence of a steroid, a saponin, a tannin, a flavanoid, a coumarin, a triterpene, and an essential oil, as indicated by the results of preliminary phytochemical screening. As triterpenoids,[28] saponins,[29] flavonoids,[28,29] and essential oil[30] from other plants have been reported to have central nervous system depressant activity, it is probable that the components that are present in abundance in the SIE might contribute, in part, for the observed central nervous system activity.

## CONCLUSION

The results of the present investigation indicate that the hydroalcoholic extract of *S. indicus* has central nervous depressant activity. The investigation also highlights the fact that anxiolytic activity of this hydroalcoholic extract of *S. indicus* was observed only at a low dose (100 mg/kg, *p.o*.). Furthermore, the results obtained in the present study suggest that *S. indicus* has anticonvulsant activity, which lends pharmacological justification to the use of the plant extract by traditional medicine practitioners in the treatment of epilepsy. Thus, this study provides experimental support for the traditional medicinal use of this plant for nervous disorders.
